# A clinical guide to assess the immune response to sepsis: from bench to bedside

**DOI:** 10.62675/2965-2774.20240179-en

**Published:** 2024-11-26

**Authors:** José Pedro Cidade, Gonçalo Guerreiro, Pedro Póvoa

**Affiliations:** 1 Centro Hospitalar Lisboa Ocidental Hospital São Francisco Xavier Department of Intensive Care Lisboa Portugal Intensive Care Unit 4, Department of Intensive Care, Hospital São Francisco Xavier, Centro Hospitalar Lisboa Ocidental - Lisbon, Portugal.

Over the last decade, there has been a remarkable expansion in our knowledge regarding the intricate pathological mechanisms underlying sepsis. This quintessential medical disorder, defined as a life-threatening, complex syndrome resulting from a dysregulated host immune response to infection, has prompted ongoing updates to diagnostic criteria and sepsis management and resulted in increased awareness among healthcare professionals. The global burden of sepsis highlights the disease's importance as a major health concern and a leading contributor to mortality and critical illness worldwide.

It is widely acknowledged that the physiological derangements observed in patients with sepsis, culminating in severe multiple organ failure, predominantly stem from a combination of conflicting inflammatory and anti-inflammatory signaling pathways. However, substantial research has shown that patients with sepsis exhibit only a transient hyperinflammation phase and swiftly transition to an immunosuppressed state.^(1)^ This biological duality has become increasingly apparent due to the increased risk of mortality (40 - 80%) and hospital-acquired infections among a significant proportion of patients with sepsis.^(2)^ This has driven the current literature to focus increasingly on the downregulated mechanisms of immune function and concurrent septic immunoparalysis. While direct and comprehensive evidence linking mortality risk, infection risk, and intensive care unit (ICU) complications to this immunosuppressed state has not been reported, increasing evidence suggests that immunoparalysis independently contributes to poor ICU prognoses and higher mortality rates. Modulating immunoparalysis may be crucial to increase survivability in severely ill patients with sepsis.

Therefore, direct assessment of immune function is crucial for accurate characterization of the immune status of patients with sepsis, who are at risk of hospital-acquired infections and ICU mortality within a heterogeneous critical population.^(3)^ Despite its importance, immune system assessment has been overlooked in the severity scoring systems commonly used in clinical practice in the ICU due in part to the lack of rigorous and standardized assessment of immune function and its integration into available scoring systems. Given the growing need to reassess the accuracy of current ICU severity indices, such as the novel Sequential Organ Failure Assessment (SOFA) 2.0, the evaluation of the immune system has emerged as a crucial factor that cannot be overlooked.^(4)^ Integrating immune system assessment into these scores may provide a more personalized and accurate depiction of patients’ illness severity, thereby enabling early organ support interventions and investigations into immune-modulating therapies to increase patients’ chances of survival. The increasing understanding of immune status dynamics in patients with sepsis emphasizes that a one-size-fits-all approach is inherently flawed, and future updates to patient evaluation methodologies should consider this to advance modern clinical practice.

However, a robust yet feasible immune system assessment at the bedside remains lacking. Due to the complexity of humoral and cellular pathways and their interactions, several monitoring techniques have been studied, focusing not only on alterations in the serum levels of innate and adaptive immunity components but also on their function. These proposed "immune organ" evaluations range from simple analytical serum markers and leukocyte (sub-)population quantifications to more complex and less-widely available functional tests (such as phagocytosis assays, *ex vivo* cellular cytokine release and cellular membrane marker expression tests, and *in vitro* immune stimulation tests).

Extensive evidence exists regarding the routine determination of inflammatory biomarkers to allow for the early recognition of critical illness in patients with sepsis and the estimation of patient prognosis. However, the clinical utility of inflammatory biomarkers cannot be overstated, and their interpretation should consider systemic clinical manifestations, organ dysfunction, and microbiological documentation.^(5)^ The CAPTAIN prospective multicenter cohort study revealed that isolated circulating biomarkers, either individually or in combination, poorly discriminate between septic and nonseptic inflammation.^(6)^ However, a recent proposal by Cajander et al. highlights that these biomarkers may be integrated into predictive algorithms, profiling patients to guide clinical decisions in the setting of sepsis.^(7)^ In addition, increasing evidence suggests the existence of tissue compartmentalization in patients with sepsis, in which the manipulation of blood immune factors does not consistently reflect alterations in tissue immune profiles. This discordant compartmentalization highlights the importance of assessing tissue-specific immune biomarker profiles to reliably predict sepsis progression and prognosis.^(8)^ Therefore, the use of the serological behavior of prognostic immune markers to guide sepsis management or antibiotic stewardship must be viewed, in principle, as valuable adjunctive information, aiding in a progressively more comprehensive and patient-tailored approach to care.

The white cell count is considered an important factor with a direct clinical impact on critically ill patients with sepsis, especially when it is considerably deranged. Due to the strong influence of the coronavirus disease 2019 (COVID-19) pandemic, leukocyte and subpopulation counts have been identified as widely available markers for diagnosing and prognosticating sepsis and can be used to identify patients with sepsis who have an increased risk of in-hospital mortality. A retrospective study of over seven thousand patients with sepsis identified persistent lymphopenia (< 1×10^9^ cells/µL) as a robust, independent risk factor for in-hospital mortality.^(9)^ These data align with several previously published studies indicating a ‘dose-response’ association between lymphopenia and an increased risk of hospitalization due to infection, healthcare-associated infections, prolonged in-hospital stay and mortality. Therefore, persistent lymphopenia can serve as an easily determinable, widely available, nearly cost-free and highly informative point-of-care immune assessment.

**Figure 1:**
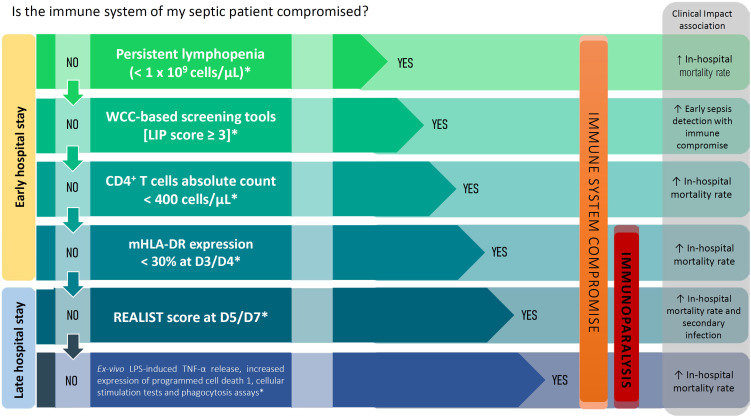
Proposed decision-making algorithm for ascertaining immune system compromise and immunoparalysis in patients with sepsis.

Furthermore, Liu et al. recently proposed an effective sepsis screening tool based on the white cell count, international normalized ratio, and procalcitonin (LIP) score, which demonstrated high sensitivity and specificity for sepsis detection, which was consistent with the Sepsis-3 criteria (LIP score ≥ 3).^(10)^ Although this tool shows high predictive value for diagnosing sepsis-related immune compromise [area under the curve (AUC) 0.974] using commonly measured clinical indicators (Figure 1S - Supplementary Material), its applicability has not been validated in patients undergoing specific treatments such as anticoagulants or glucocorticoids, and it performs less accurately in patients with sepsis originating from pulmonary infections (AUC 0.946). However, these laboratory tools hold promise for rapid and widespread implementation, particularly in low- and middle-income countries. In contrast, Polilli et al. reported that an absolute number of <400/µL CD4^+^ T cells at admission was an independent predictor of mortality in patients with sepsis.^(11)^ In a more labor-intensive but well-designed study, Jundi et al. concluded that serial assessments of leukocyte function were more predictive of the clinical course than leukocyte parameterization was, providing important insights into the already known apoptosis-induced lymphopenia contributing to sepsis-associated immunosuppression.^(12)^

More recently, persistently reduced expression of the monocytic human leukocyte antigen-DR isotype (mHLA-DR) has emerged as a universal biomarker associated with immunoparalysis in sepsis, although its assessment is labor intensive and not widely available. Several studies have verified that mHLA-DR expression via flow cytometry (< 30%) at ICU admission, and over time, was significantly associated with increased ICU mortality and independently predictive of the development of hospital-acquired infections.^(13)^ Moreover, the REALIST study demonstrated that the cumulative percentage of immature neutrophils (CD10− CD16− ≥ 23.5%), in conjunction with mHLA-DR (≤ 7627 Ab/C) and serum interleukin-10 (IL-10) (≥ 8.5pg/mL) levels measured on the fifth to seventh day after admission, can reliably identify subgroups of patients at high risk of developing ICU-acquired infections.^(14)^ This approach provides a potential diagnostic tool for identifying high-risk patients in the ICU and optimizing their care and trial enrichment.

More laborious and advanced techniques, including ex vivo lipopolysaccharide-induced tumor necrosis factor-α release, increased expression of immune checkpoint molecules [such as programmed cell death 1 (PD-1)], cellular stimulation tests, and phagocytosis assays, represent immune system evaluations with potential clinical implications.^(15)^ However, their availability is limited, and validated and standardized protocols for prompt application at the patient's bedside are still lacking. As these methods require specialized equipment and technical expertise, their integration into routine clinical practice remains challenging, especially in low- and lower-middle-income countries (LMICs).

Building upon these findings, several immunomodulatory therapies have been proposed, aiming to provide anti-inflammatory strategies to dampen an overactive immune response [such as IL-1 blockade^(16)^ or IF-γ 1b^(17)^] or to counteract prominent septic immunoparalysis [including anti-PD-L1,^(18)^ glycosylated recombinant human IL-7 (CYT107) or granulocyte-macrophage colony-stimulating factor (GM-CSF)^(19)^]. However, the most recent randomized controlled trials testing these molecules have yielded no significant discernible benefit in terms of patient outcomes or survival. The main obstacle to these molecules may be the heterogeneity of the sepsis immune response, necessitating the identification of specific therapeutic agents tailored to the pathogen culprit and immune profile, targeting time- and individual-dependent immunotherapy.^(20)^

Integrating immune system assessment with clinical scores has the potential to provide a more personalized and accurate depiction of patients’ illness severity, thereby enabling early organ support interventions and immune-modulating therapy investigations to increase patients’ chances of survival. Therefore, we have developed a decision algorithm that attempts to ascertain immune system compromise and immunoparalysis in patients with sepsis (Figure 1 and Figure 1S and Table 1S - Supplementary Material). Although speculative and not yet validated, this algorithm represents a preliminary attempt to provide physicians with a structured approach to assess patients’ immune systems. It includes several evidence-based techniques that can be used in clinical scenarios with scarce resources, making it globally accessible and usable at the patient bedside.

The algorithm encompasses various strategies to assess patients’ immune systems. While some have not yet been validated to reliably indicate immunoparalysis, they have been significantly associated with a compromised immune system in patients with sepsis, and some are widely available for implementation as soon as patients are admitted to the emergency room or hospital. The current evidence regarding the risk of immune system compromise or paralysis is summarized in table 1. * See Figure 1S (Supplementary Material). WCC - white cell count; LIP - sepsis screening tool based on the white cell count, international normalized ratio, and procalcitonin; mHLA-DR - monocytic human leukocyte antigen-DR isotype; D - day; TNF-α - tumor necrosis factor alpha; LPS - lipopolysaccharide.

The expanding understanding of immune status dynamics in patients with sepsis makes it clear that future updates to patient evaluation methodologies and therapies should utilize a patient-tailored approach. This approach will advance modern clinical practice and help physicians treating severely ill patients adjust their therapeutic plans to specific patient profiles.
