# Supplementary material for: A clinical guide to assess the immune response to sepsis: from bench to bedside
Source: Crit Care Sci. 2024 Nov 26;36:e20240179en. doi: 10.62675/2965-2774.20240179-en (PMC11634233; doi:10.62675/2965-2774.20240179-en)
Supplement: Supplementary file 1 [file 2965-2774-ccsci-36-e20240179en-suppl01.pdf]

Critical Care Science
https://doi.org/10.62675/2965-2774.20240179-en

SUPPLEMENTARY MATERIAL

A clinical guide to assess the immune response to sepsis: from bench to bedside

José Pedro Cidade[1], Gonçalo Guerreiro[1], Pedro Póvoa[1]

– LIP scoring system

Itens	Score		
	0	1	2
Lymphocyte count (*10 ^ 9/L)	> 1	0.7 - 1	< 0.7
International normalized ratio (INR)	< 1.2	1.2 - 1.4	> 1.4
Procalcitonin (PCT, ng/mL)	< 0.5	0.5 - 2	> 2

Source: Liu B, Du H, Zhang J, Jiang J, Zhang X, He F, et al. Developing a new sepsis screening tool based on lymphocyte count, international normalized ratio and procalcitonin (LIP score). Sci Rep. 2022;12(1):20002.[1]
Article is licensed under a Creative Commons Attribution 4.0 International License. No changes were made.

– $CD4^+$ T cells absolute count < 400 cells/µL.[2]

– Persistent lymphopenia (< $1x10^9$ cells/µL) at D3/D4 of hospital length of stay.[3]

– Monocytic HLD-DR expression < 30% at D3/D4 of hospital length of stay.[4,5]

– REALIST score at D5/D7 of hospital length of stay

mHLA-DR
≤ 7627 Ab/C
1 point

Immature neutrophils
≥ 23.5 %
1 point

IL-10
≥ 8.5 pg/ml
1 point

Helps in assessing the risk of secondary infection

REALIST score	Secondary infection at D30	
	Incidence	Adjusted HR
0	8.2%	–
1	11.9%	1.05 (0.30–3.64)
2	30.8%	2.33 (0.81–6.73)
3	46.0%	3.22 (1.09–9.50)

Source: Tremblay JA, Peron F, Kreitmann L, Textoris J, Brengel-Pesce K, Lukaszewicz AC, Quemeneur L, Vedrine C, Tan LK, Venet F, Rimmele T, Monneret G; REALISM study group. A stratification strategy to predict secondary infection in critical illness-induced immune dysfunction: the REALIST score. Ann Intensive Care. 2022;12(1):76.[6]
Article is licensed under a Creative Commons Attribution 4.0 International License. No changes were made.

– *Ex-vivo* LPS-induced TNF-α release, increased expression of programmed cell death 1 (PD-1), cellular stimulation tests and phagocytosis assays[7,8]

– *Ex-vivo* LPS-induced TNF-α release, increased expression of programmed cell death 1 (PD-1), cellular stimulation tests and phagocytosis assays[7,8]

Figure 1S - Decision algorithm to ascertain immune system compromise and immunoparalysis in patients with sepsis.
HLA-DR - human leukocyte antigen-DR isotype; D3/D4 and D5/D7 - Day 3, 4, 5 or 7 after hospital admission, respectively; IL – interleukin; LPS – lipopolysaccharide; TNF-α - tumor necrosis factor-α.

This is an open access article under the CC BY license (https://creativecommons.org/licenses/by/4.0/).

Table 1S - Current evidence on the proposed algorithm variables to assess immune system compromise and immunoparalysis in patients with sepsis

Algorithm variable	Evidence
Persistent lymphopenia (<1x10^9 cells/μL)	Persistent lymphopenia (< 1x10^9 cells/μL) was an independent risk factor for in-hospital death (HR for death of 1.89 (95%CI 1.32 - 2.71; p < 0.001)[2]
CD4$^+$ T cells absolute count < 400 cells/μL	CD4$^+$ T cells absolute count < 400 cells/μL was an independent predictor of in-hospital mortality (aOR 5.3; 95%CI 1.65 - 17.00; p = 0.005)[4]
Monocytic HLA-DR expression < 30% at D3/D4	Monocytic HLA-DR expression < 3 0% at D3/D4 was independently associated with in-hospital mortality (aOR 6.48; 95%CI 1.62 - 25.93; p = 0.008)[7]
LIP score ≥ 3	Sepsis detection with immune system compromise (AUROC 0.974; 95%CI 0.961 - 0.983). Sensitivity 92.8%, specificity 94.1%, PPV 94.1%, NPV 92.9%)[9]
REALIST score at D5/D7	REALIST score was independently associated with increased risk of secondary infection (22.2% of events, adjusted HR 3.22 (95%CI 1.09 - 9.50; p = 0.034) and mortality (5.3% of events; p = 0.001)[10]
Ex vivo lipopolysaccharide-induced tumor necrosis factor-α release, increased expression of immune checkpoint molecules [such as programmed cell death 1 (PD-1)], cellular stimulation tests and phagocytosis assays	*Ex vivo* Lipopolysaccharide-induced tumor necrosis factor-α release (iTNF) demonstrated good correlation with mHLA-DR expression in septic patients (correlation coefficient 0.7; p = 0.003)[11]<br>Combination of PD-1$^+$ CD4$^-$ CD8$^-$ T and monocytic HLA-DR$^+$ expressions revealed a good ability to predict mortality of sepsis patients (AUROC = 0.921; 95%CI 0.762 - 0.987)[11]<br>Altered neutrophil chemotaxis and phagocytosis, and an increased number of circulating immature granulocytes were associated with an increased risk of death after septic shock (OR 1.52; 95%CI 1.003 - 2.307; p = 0.048)[11]

HR - hazard ratio; 95%CI - 95% confidence interval; aOR - adjusted odds ratio; LIP - lymphocyte count, international normalized ratio, and procalcitonin; HLA-DR - human leukocyte antigen-DR isotype; D3/D4 and D5/D7 - Day 3, 4, 5 or 7 after hospital admission, respectively; AUROC - area under receiver operating characteristic curve; PPV - positive predictive value; NPV - negative predictive value.

REFERENCES

1. Liu B, Du H, Zhang J, Jiang J, Zhang X, He F, et al. Developing a new sepsis screening tool based on lymphocyte count, international normalized ratio and procalcitonin (LIP score). Sci Rep. 2022;12(1):20002.
2. Polilli E, Esposito JE, Frattari A, Trave F, Sozio F, Ferrandu G, et al. Circulating lymphocyte subsets as promising biomarkers to identify septic patients at higher risk of unfavorable outcome. BMC Infect Dis. 2021;21(1):780.
3. Adigbli D, Liu R, Meyer J, Cohen J, Di Tanna GL, Gianacas C, et al. Early persistent lymphopenia and risk of death in critically ill patients with and without sepsis. Shock. 2024;61(2):197-203.
4. Monneret G, Lepape A, Voirin N, Bohé J, Venet F, Debard AL, et al. Persisting low monocyte human leukocyte antigen-DR expression predicts mortality in septic shock. Intensive Care Med. 2006;32(8):1175-83.
5. Landelle C, Lepape A, Voirin N, Tognet E, Venet F, Bohé J, et al. Low monocyte human leukocyte antigen-DR is independently associated with nosocomial infections after septic shock. Intensive Care Med. 2010;36(11):1859-66.
6. Tremblay JA, Peron F, Kreitmann L, Textoris J, Brengel-Pesce K, Lukaszewicz AC, Quemeneur L, Vedrine C, Tan LK, Venet F, Rimmele T, Monneret G; REALISM study group. A stratification strategy to predict secondary infection in critical illness-induced immune dysfunction: the REALIST score. Ann Intensive Care. 2022;12(1):76.
7. Pfortmueller CA, Meisel C, Fux M, Schefold JC. Assessment of immune organ dysfunction in critical illness: utility of innate immune response markers. Intensive Care Med Exp. 2017;5(1):49.
8. Guignant C, Lepape A, Huang X, Kherouf H, Denis L, Poitevin F, et al. Programmed death-1 levels correlate with increased mortality, nosocomial infection and immune dysfunctions in septic shock patients. Crit Care. 2011;15(2):R99.
9. Jundi B, Ryu H, Lee DH, Abdulnour RE, Engstrom BD, Duvall MG, et al. Leukocyte function assessed via serial microlitre sampling of peripheral blood from sepsis patients correlates with disease severity. Nat Biomed Eng. 2019;3(12):961-73.
10. Fisher CJ Jr, Dhainaut JF, Opal SM, Pribble JP, Balk RA, Slotman GJ, et al. Recombinant human interleukin 1 receptor antagonist in the treatment of patients with sepsis syndrome. Results from a randomized, double-blind, placebo-controlled trial. Phase III rhIL-1ra Sepsis Syndrome Study Group. JAMA. 1994;271(23):1836-43.
11. Roquilly A, Francois B, Huet O, Launey Y, Lasocki S, Weiss E, Petrier M, Hourmant Y, Bouras M, Lakhal K, Le Bel C, Flattres Duchaussoy D, Fernández-Barat L, Ceccato A, Flet L, Jobert A, Poschmann J, Sebille V, Feuillet F, Koulenti D, Torres A; Atlanrea study group and the Société Française d'Anesthésie Réanimation (SFAR) Research Network. Interferon gamma-1b for the prevention of hospital-acquired pneumonia in critically ill patients: a phase 2, placebo-controlled randomized clinical trial. Intensive Care Med. 2023;49(5):530-44.
