# Supplementary material for: A clinical guide to assess the immune response to sepsis: from bench to bedside
Source: Crit Care Sci. 2024 Nov 26;36:e20240179en. doi: 10.62675/2965-2774.20240179-en (PMC11634233; doi:10.62675/2965-2774.20240179-en)
Supplement: Translation

# Translation: Um guia clínico para avaliar a resposta imunológica à sepse: da bancada ao leito

**DOI:** 10.62675/2965-2774.20240179-pt

**Authors:** José Pedro Cidade, Gonçalo Guerreiro, Pedro Póvoa

**Affiliations:** 1 Lisboa Portugal Unidade de Terapia Intensiva 4, Departamento de Cuidados Intensivos, Hospital São Francisco Xavier, Centro Hospitalar Lisboa Ocidental - Lisboa, Portugal

Na última década, houve uma expansão notável em nosso conhecimento sobre os intrincados mecanismos patológicos subjacentes à sepse. Esse distúrbio médico, por excelência definido como uma síndrome complexa com risco de morte, resultante de uma resposta imunológica desregulada do hospedeiro à infecção, levou a atualizações contínuas dos critérios de diagnóstico e do manejo da sepse e resultou em uma maior conscientização dos profissionais de saúde. O ônus global da sepse destaca a relevância da doença como um importante problema de saúde e um dos principais fatores que contribuem para a mortalidade e doenças graves em todo o mundo.

Reconhece-se em grande medida que os distúrbios fisiológicos observados em pacientes com sepse, que culminam em falência grave de múltiplos órgãos, decorrem predominantemente de uma combinação de vias de sinalização inflamatórias e anti-inflamatórias conflitantes. No entanto, pesquisas substanciais demonstraram que os pacientes com sepse exibem apenas uma fase transitória de hiperinflamação e passam rapidamente para um estado imunossuprimido.^(1)^ Essa dualidade biológica tornou-se cada vez mais aparente devido ao aumento do risco de mortalidade (40 a 80%) e de infecções hospitalares em uma proporção significativa de pacientes com sepse.^(2)^ Isso fez com que a literatura atual se concentrasse cada vez mais nos mecanismos desregulados da função imunológica e na imunoparalisia séptica concomitante. Embora não tenham sido relatadas evidências diretas e abrangentes que relacionem o risco de mortalidade, o risco de infecção e as complicações na unidade de terapia intensiva (UTI) a esse estado imunossuprimido, cada vez mais evidências sugerem que a imunoparalisia contribui independentemente para prognósticos desfavoráveis na UTI e taxas de mortalidade mais altas. A modulação da imunoparalisia pode ser crucial para aumentar a capacidade de sobrevida em pacientes críticos com sepse.

Assim, a avaliação direta da função imunológica é crucial para a caracterização precisa do estado imunológico de pacientes com sepse, que correm risco de infecções hospitalares e mortalidade na UTI no âmbito de uma população crítica heterogênea.^(3)^ Apesar de sua importância, a avaliação do sistema imunológico tem sido negligenciada nos sistemas de pontuação de gravidade comumente usados na prática clínica na UTI devido, em parte, à falta de uma avaliação rigorosa e padronizada da função imunológica e sua integração nos sistemas de pontuação disponíveis. Dada a crescente necessidade de reavaliar a precisão dos atuais índices de gravidade da UTI, como o novo *Sequential Organ Failure Assessment* (SOFA) 2.0, a avaliação do sistema imunológico surgiu como um fator crucial que não pode ser ignorado.^(4)^ A integração da avaliação do sistema imunológico a esses escores pode fornecer uma descrição mais personalizada e precisa da gravidade da doença dos pacientes, permitindo intervenções precoces de suporte a órgãos e investigações sobre terapias imunomoduladoras para aumentar as chances de sobrevida dos pacientes. A compreensão cada vez maior da dinâmica do estado imunológico em pacientes com sepse enfatiza que uma abordagem padronizada para todos é inerentemente falha. Futuras atualizações das metodologias de avaliação de pacientes devem levar isso em consideração para o avanço da prática clínica moderna.

No entanto, ainda não há uma avaliação robusta e viável do sistema imunológico à beira do leito. Devido à complexidade das vias humoral e celular e suas interações, várias técnicas de monitoramento foram estudadas, com foco não apenas nas alterações dos níveis séricos dos componentes da imunidade inata e adaptativa, mas também em sua função. Essas avaliações de "órgãos imunológicos" propostas variam de simples marcadores séricos analíticos e quantificações de (sub)populações de leucócitos a testes funcionais mais complexos e menos disponíveis (como ensaios de fagocitose, liberação de citocinas celulares *ex vivo* e testes de expressão de marcadores de membrana celular e testes de estimulação imunológica *in vitro*).

Há inúmeras evidências da determinação rotineira de biomarcadores inflamatórios para permitir o reconhecimento precoce de doenças críticas em pacientes com sepse e a estimativa do prognóstico do paciente. No entanto, a utilidade clínica dos biomarcadores inflamatórios não pode ser exagerada, e sua interpretação deve considerar as manifestações clínicas sistêmicas, a disfunção orgânica e a documentação microbiológica.^(5)^ O estudo de coorte multicêntrico prospectivo CAPTAIN revelou que biomarcadores circulantes isolados, individualmente ou combinados, discriminam mal entre inflamação séptica e não séptica.^(6)^ No entanto, uma proposta recente de Cajander et al. destaca que esses biomarcadores podem ser integrados em algoritmos preditivos, traçando o perfil dos pacientes para orientar as decisões clínicas no cenário da sepse.^(7)^ Além disso, cada vez mais evidências sugerem a existência de compartimentalização tecidual em pacientes com sepse, na qual a manipulação de fatores imunológicos sanguíneos não reflete consistentemente alterações nos perfis imunológicos teciduais. Essa compartimentalização discordante destaca a importância de avaliar os perfis de biomarcadores imunológicos específicos do tecido para prever com segurança a progressão e o prognóstico da sepse.^(8)^ Desse modo, o uso do comportamento sorológico de marcadores imunológicos prognósticos para orientar o tratamento da sepse ou a administração de antibióticos deve ser visto, em princípio, como uma informação adjuvante valiosa, auxiliando em uma abordagem progressivamente mais abrangente e personalizada do paciente.

A contagem de leucócitos (CL) é considerada um fator importante com impacto clínico direto em pacientes graves com sepse, especialmente quando está consideravelmente alterada. Devido à forte influência da pandemia da doença causada pelo coronavírus 2019 (COVID-19), as CLs e subpopulações foram identificadas como marcadores amplamente disponíveis no diagnóstico e no prognóstico da sepse e podem ser usadas para identificar pacientes com sepse que têm um risco maior de mortalidade hospitalar. Um estudo retrospectivo de mais de sete mil pacientes com sepse identificou a linfopenia persistente (< 1×10^9^ células/µL) como um fator de risco robusto e independente de mortalidade hospitalar.^(9)^ Esses dados se alinham a vários estudos já publicados que indicam uma associação de "dose-resposta" entre linfopenia e um risco maior de hospitalização por infecção, infecções associadas à assistência médica, internação hospitalar prolongada e mortalidade. Portanto, a linfopenia persistente pode servir como uma avaliação imunológica facilmente determinável, amplamente disponível, quase sem custos e altamente informativa no local de assistência.

Além disso, Liu et al. propuseram recentemente uma ferramenta eficaz de triagem de sepse com base na CL, na razão normalizada internacional e na procalcitonina, o escore LIP, que demonstrou alta sensibilidade e especificidade na detecção de sepse, o que foi consistente com os critérios da Sepse-3 (escore LIP ≥ 3).^(10)^ Embora essa ferramenta demonstre alto valor preditivo no diagnóstico de comprometimento imunológico relacionado à sepse [área sob a curva (AUC) de 0,974] por meio de indicadores clínicos comumente medidos (Figura 1S - Material Suplementar), sua aplicabilidade não foi validada em pacientes submetidos a tratamentos específicos, como anticoagulantes ou glicocorticoides; e seu desempenho é menos preciso em pacientes com sepse originada de infecções pulmonares (AUC de 0,946). No entanto, essas ferramentas laboratoriais são promissoras na implementação rápida e generalizada, principalmente em países de baixa e média renda. Em contraste, Polilli et al. relataram que um número absoluto de células T CD4+ <400/µL na admissão foi um preditor independente de mortalidade em pacientes com sepse.^(11)^ Em um estudo mais laborioso, porém bem desenhado, Jundi et al. concluíram que as avaliações seriadas da função leucocitária eram mais preditivas do curso clínico do que a parametrização de leucócitos, fornecendo informações importantes sobre a já conhecida linfopenia induzida por apoptose, a qual contribui para a imunossupressão associada à sepse.^(12)^

Mais recentemente, a expressão persistentemente reduzida do isótipo do antígeno leucocitário humano-DR monocítico (mHLA-DR) surgiu como um biomarcador universal associado à imunoparalisia na sepse, embora sua avaliação seja trabalhosa e não esteja amplamente disponível. Vários estudos verificaram a expressão de mHLA-DR por citometria de fluxo (< 30%) na admissão na UTI e, ao longo do tempo, essa expressão foi significativamente associada ao aumento da mortalidade na UTI e preditiva independentemente do desenvolvimento de infecções hospitalares.^(13)^ Além disso, o estudo REALIST demonstrou que a proporção cumulativa de neutrófilos imaturos (CD10- CD16- ≥ 23.5%), em conjunto com o mHLA-DR (≤ 7.627 Ab/C) e os níveis séricos de interleucina 10 (IL-10) (≥ 8,5pg/mL), medidos do quinto ao sétimo dia após a admissão, pode identificar de forma confiável subgrupos de pacientes com alto risco de desenvolver infecções adquiridas na UTI.^(14)^ Essa abordagem fornece uma ferramenta de diagnóstico em potencial para identificar pacientes de alto risco na UTI e otimizar sua assistência e enriquecimento de estudos.

Técnicas mais laboriosas e avançadas, incluindo a liberação de fator de necrose tumoral alfa induzida por lipopolissacarídeo *ex vivo*, aumento da expressão de moléculas de ponto de verificação imunológico [como a morte celular programada 1 (PD-1)], testes de estimulação celular e ensaios de fagocitose, representam avaliações do sistema imunológico com possíveis implicações clínicas.^(15)^ No entanto, sua disponibilidade é limitada, e ainda faltam protocolos validados e padronizados para aplicação imediata no leito do paciente. Como esses métodos exigem equipamentos especializados e conhecimento técnico, sua integração à prática clínica de rotina continua sendo um desafio, especialmente em países de baixa e média renda.

Apoiando-se nessas descobertas, várias terapias imunomoduladoras foram propostas, com o objetivo de fornecer estratégias anti-inflamatórias para atenuar uma resposta imunológica hiperativa [como o bloqueio de IL-1^(16)^ ou IF-γ 1b^(17)^] ou para neutralizar a imunoparalisia séptica proeminente [incluindo anti-PD-L1,^(18)^ IL-7 humana recombinante glicosilada (CYT107) ou fator estimulador de colônias de granulócitos e macrófagos (GM-CSF)^(19)^]. No entanto, os estudos controlados e randomizados mais recentes que testaram essas moléculas não produziram benefício significativo perceptível, em termos de desfechos ou sobrevida dos pacientes. O principal obstáculo para essas moléculas pode ser a heterogeneidade da resposta imunológica da sepse, o que exige a identificação de agentes terapêuticos específicos adaptados ao patógeno responsável e ao perfil imunológico, visando à imunoterapia dependente do tempo e do indivíduo.^(20)^

A integração da avaliação do sistema imunológico com os escores clínicos tem o potencial de fornecer uma descrição mais personalizada e precisa da gravidade da doença dos pacientes, permitindo intervenções precoces de suporte a órgãos e investigações de terapia imunomoduladora para aumentar as chances de sobrevida dos pacientes. Portanto, desenvolvemos um algoritmo de decisão que tenta determinar o comprometimento do sistema imunológico e a imunoparalisia em pacientes com sepse (Figura 1, Figura 1S e Tabela 1S - Material Suplementar). Embora especulativo e ainda não validado, esse algoritmo representa uma tentativa preliminar de fornecer aos médicos uma abordagem estruturada para avaliar o sistema imunológico dos pacientes. Ele inclui várias técnicas baseadas em evidências que podem ser usadas em cenários clínicos com recursos escassos, tornando-o globalmente acessível e utilizável no leito do paciente.

**Figura 1:** Proposta de algoritmo de tomada de decisão para verificar o comprometimento do sistema imunológico e a imunoparalisia em pacientes com sepse.

O algoritmo engloba várias estratégias para avaliar o sistema imunológico dos pacientes. Embora algumas ainda não tenham sido validadas para indicar de forma confiável a imunoparalisia, elas foram significativamente associadas a um sistema imunológico comprometido em pacientes com sepse, e algumas estão amplamente disponíveis para implementação assim que os pacientes são admitidos no pronto-socorro ou no hospital. As evidências atuais sobre o risco de comprometimento ou paralisia do sistema imunológico estão resumidas na tabela 1. * Vide a Figura 1S (Material Suplementar). CL - contagem de leucócitos; LIP - ferramenta de triagem de sepse com base na contagem de leucócitos, na razão normalizada internacional e na procalcitonina; mHLA-DR - antígeno leucocitário humano-DR monocítico; D - dia; TNF-α - fator de necrose tumoral alfa; LPS – lipopolissacarídeo.

A compreensão cada vez maior da dinâmica do estado imunológico em pacientes com sepse deixa claro que as futuras atualizações das metodologias e terapias de avaliação do paciente devem utilizar uma abordagem adaptada ao paciente. Essa abordagem avançará a prática clínica moderna e ajudará os médicos que tratam de pacientes críticos a ajustar seus planos terapêuticos a perfis específicos de pacientes.
